# The Immune Correlates of *Orthohantavirus* Vaccine

**DOI:** 10.3390/vaccines9050518

**Published:** 2021-05-18

**Authors:** Joon-Yong Bae, Jin Il Kim, Mee Sook Park, Gee Eun Lee, Heedo Park, Ki-Joon Song, Man-Seong Park

**Affiliations:** Department of Microbiology, Institute for Viral Diseases, Biosafety Center, Korea University College of Medicine, Seoul 02841, Korea; harbe3103@korea.ac.kr (J.-Y.B.); jinil_kim@korea.ac.kr (J.I.K.); parkmees@korea.ac.kr (M.S.P.); geeeun950606@gmail.com (G.E.L.); phd0919@naver.com (H.P.); songmicr@korea.ac.kr (K.-J.S.)

**Keywords:** antigenicity, glycoprotein, *orthohantavirus*, pandemic, vaccine

## Abstract

Zoonotic transmission of *orthohantaviruses* from rodent reservoirs to humans has been the cause of severe fatalities. Human infections are reported worldwide, but vaccines have been approved only in China and Korea. *Orthohantavirus* vaccine development has been pursued with no sense of urgency due to the relative paucity of cases in countries outside China and Korea. However, the *orthohantaviruses* continuously evolve in hosts and thus the current vaccine may not work as well against some variants. Therefore, a more effective vaccine should be prepared against the *orthohantaviruses*. In this review, we discuss the issues caused by the *orthohantavirus* vaccine. Given the pros and cons of the *orthohantavirus* vaccine, we suggest strategies for the development of better vaccines in terms of pandemic preparedness.

## 1. Introduction

*Hantaan orthohantavirus (HTNV)* is a member of the *Hantaviridae* family, which is characterized by a single-stranded negative-sense RNA genome consisting of three RNA segments designated as small (S), medium (M), and large (L). The nucleocapsid protein (N), membrane surface glycoproteins (GPs: Gn and Gc) and RNA-dependent RNA polymerase protein (RdRp) are expressed from the S, M, and L segments, respectively. HTNV expresses only N, GPs, and RdRp from the negative-sense RNA genome. In addition, some hantaviruses express non-structural protein (NSs) not from an ambisense strategy but from an open reading frame (ORF) within the N gene [1]. HTNV was first detected in the field mouse, *Apodemus agrarius*, in 1976 and isolated by inoculating *A. agrarius* with acute-phase patient sera in 1978 [2,3]. HTNV was assigned to the genus *hantavirus* with a different mode of transmission and unique terminal genome sequence [4].

So far, more than 20 species of *orthohantaviruses* have been identified [5]. Each species tends to be carried by a specific rodent or insectivore host [6]. The number of *orthohantavirus* species might increase as new species are discovered [7]. For most *orthohantaviruses*, human pathogenicity is unknown [8]. *Orthohantaviruses* associated with human disease have been isolated all from rodent species [9]. Human pathogenic *orthohantaviruses* have shown three main geographic distributions: HTNV and *Seoul orthohantavirus* (SEOV) in the Far East, *Puumala orthohantavirus* (PUUV) and *Dobrava-Belgrade orthohantavirus* (DOBV) in Europe and Russia, and *Sin Nombre* (SNV) and *Andes orthohantavirus* (ANDV) in the Americas [6]. Human infection cases tend to be geographically distinct due to rodent host specificity and distribution [10]. However, only SEOV human infections have been reported globally due to the worldwide distribution of its reservoir host the Norway rat (*Rattus norvegicus*) [11,12,13].

Human *orthohantavirus* infection is, in most cases, the result of zoonotic transmission through inhalation of the aerosolized viral particles from the excretions of infected rodents [14]. Although there have been no reports of human-to-human transmission of *orthohantavirus* from patients with hemorrhagic fever with renal syndrome (HFRS), there have been reports of human-to-human transmission of ANDV from patients with hantavirus (cardio)-pulmonary syndrome (HPS or HCPS) through close contacts or nosocomial transmission most likely involving respiratory secretions, saliva, or both [15,16,17,18]. Zoonotic viruses causing high case fatalities in humans, especially with instances of human-to-human transmission, might be worth watching for emerging pandemic or bioterrorism threats [14,19,20].

Different species of human pathogenic *orthohantaviruses* cause different symptoms with different case fatality rates. The so-called Old World *orthohantaviruses* HTNV, SEOV, PUUV, and DOBV cause HFRS with a case fatality rate from <1–15%. HTNV and DOBV are associated with severe, SEOV with moderate, and PUUV with mild cases. The New World *orthohantaviruses* SNV and ANDV cause HPS or HCPS with a case fatality rate up to 40% [6,9,15]. The *orthohantavirus* infects endothelial cells via a_v_b_3_ integrins. The a_v_b_3_ integrins regulate permeabilizing responses of vascular endothelial growth factor (VEGF) directed by VEGFR2 receptors. The accumulation of viral particles on the endothelial cell surface via interaction with a_v_b_3_ integrins is associated with hyperpermeability of endothelial cells and platelet inactivation, leading to hemorrhage [21]. However, acute *orthohantavirus* infection illness is likely also related to immune pathology [10,22]. Suboptimal amounts of neutralizing antibodies could incur the antibody-dependent enhancement of *orthohantavirus* infection in the acute phase [23]. Acute phase IgM and complement-mediated immunopathology might not be ruled out [24]. Complement receptor gC1qR/p32 has been reported to bind HTNV [25], although the involvement of this interaction in the complement pathway and HFRS has not been characterized. *Orthohantavirus* infection is asymptomatic and persistent in its natural rodent reservoirs [9]. Persistent infection in these hosts is made possible by low immune responses, whereas infection in the non-reservoir host is met with a high level of host antibody responses to clear the infection [10,26,27].

After gaining attention during the Korean War, Korean hemorrhagic fever (KHF), later designated as HFRS, had been an important military problem [3]. Such urgency prompted the development of the first vaccine and commercialization as Hantavax in Korea in 1990, just 12 years after the first isolation of HTNV from patients. China is the only other country with approved vaccines against HTNV and SEOV [28], which is related to the fact that more than 90% of the world’s total cases of HFRS mainly from HTNV or SEOV infection have been reported in China [29]. While these inactivated whole virus vaccines are still being clinically evaluated, DNA vaccines are also under development [15,29,30,31].

In terms of therapeutic countermeasures against *orthohantavirus*, there are no approved antiviral drugs. Treatment with nucleoside analog-based classical broad-spectrum antiviral drugs such as ribavirin has been attempted, but efficacy has not been proven [29,32].

## 2. Antigenicity of *Orthohantavirus* Vaccine

### 2.1. Clinical Studies for Hantavax Vaccine

Hantavax is an *orthohantavirus* vaccine licensed for use in Korea. This vaccine is made with a formalin-inactivated HTNV strain (ROK 84-105) grown in the suckling mouse brain [33]. Four clinical studies have been conducted to investigate antibody responses with different study protocols (different study populations and vaccination schedules) [33,34,35,36]. A plaque reduction neutralizing test (PRNT) was performed, and above-certain values of the reciprocal of the end-point dilution that resulted in 50% reduction in plaque number (PRNT_50_) were adopted as a criterion for neutralizing antibody (nAb) positivity. Immunofluorescence assay (IFA) and enzyme-linked immunosorbent assay (ELISA) were also performed in all studies except for Sohn et al. (2001) [34]. In these studies, the overall trend of high antibody positivity rates using IFA and ELISA and relatively low nAb positivity rate using PRNT_50_ titer were determined. The results of these studies are summarized in Table 1.

Antibody response positivity was determined one month after intramuscular injection of Hantavax [33,34] and at two and four months after [36] or one year, two years, and three years after the last injection [35]. Cho et al. [33] showed 75% nAb positivity after two consecutive doses at one-month intervals (primary doses), but the study population was small, and the results were not reproduced elsewhere. Other studies showed nAb positivity rates between 23% and 41% [34,35,36]. Whether two or three primary doses are administered, the nAb positivity rate declined to close to pre-vaccination levels after one year. Booster vaccination at that point restored nAb positivity to 45–50% in cases that received two primary doses [33,35] and 56% after three primary doses [36]. While nAb antibody responses of two primary doses + booster dose (2 + 1) were sustained fairly well one year after the booster [35], those of three primary doses + booster dose (3 + 1) declined to half of the initial booster response only three months later [36]. This difference could be attributable to differences between vaccine batches or an unknown immunological mechanism that differentiates the three from the two primary doses in the long term. Antibody responses from the 3 + 1 Hantavax vaccination schedule appear not to dwarf those from the 2 + 1 schedule. However, three primary doses giving 72% nAb positivity one month after the last primary dose compared with two primary doses giving 41% positivity showed a way for enhanced short-term protection, which might be critical for cutting the chain of infections in a pandemic situation [37].

### 2.2. Evaluation of Hantavax Vaccine Effectiveness

Field studies to evaluate the protective effect of Hantavax against HFRS were conducted in the Army of the Republic of Korea (ROK Army) [31,38]. The ROK Army’s vaccination program adopted a ‘2 + 1’ schedule, and case-control studies for HFRS and non-HFRS patients were conducted. Jung et al. [31] analyzed patients within six years and found adjusted vaccine effectiveness (VE) was 58.9%. Interestingly, the multiple-dose schedule was not better than the single-dose, which was counterintuitive. Another study [38] analyzed patients within two years and showed that Hantavax VE estimates rose from 25% for one dose to 46% for two doses to 75% for three doses. Park’s study [38] evaluated short-term Hantavax VE beginning three weeks after the first dose (average 7.3 months from the third dose). Jung [31] did not articulate the case range of time from the last dose, but the second dose to onset was a mean 289.8 days (SD 139.1), suggesting a wide range of time after the last dose. According to Song et al. [35] (Table 1), the antibody responses of the booster dose could not have waned much for Park et al.’s three-dose group [38]. Although the protective effect of 2 + 1 doses of Hantavax appears to be dose-dependent in the short term, the results were not statistically significant due to the small number of study participants.

Hantavax VEs from field studies appear moderate. However, a VE of 58.9% for participants vaccinated with only one or two doses (84%) with a wide range of time from the last dose [31] and a VE of 75% within one year after 2 + 1 vaccination appears higher than expected based on the nAb positivity rate using PRNT_50_ (Table 1). Although controversial, even higher VE has been observed. A field study conducted in Yugoslavia by a team, including the developer of Hantavax, reported 20 confirmed HFRS cases among 2000 nonvaccinated controls while no cases were observed among 1900 Hantavax vaccinated individuals [38]. The nAb positivity rate using PRNT_50_ might not reflect the correct in vivo protective responses of antibodies after the Hantavax vaccination.

### 2.3. Antigenicity of Orthohantaviruses

As summarized in Table 1, the seroconversion rates after the Hantavax vaccination using IFA or ELISA were much higher than those using PRNT. A large proportion of antibodies generated by the Hantavax vaccination is non-nAb. Choi et al. [39] showed that sera from Hantavax-immunized mice immunoprecipitated a proportionately higher amount of N than Gn and barely any Gc. It is unknown whether this antibody response is limited only to the mouse. We can only glimpse Hantavax antigenicity in humans from the study by Cho et al. [33], where the immunoblot analysis of Hantavax-vaccinated human sera showed antibodies against N and Gc. However, the data were not shared, and the conflicting observation of Gn- and Gc-specific antibodies could have been due to methodological differences in antigen recognition [33,39]. Strong HTNV N-specific IgG detection from Korean HFRS patient sera using ELISA and western blot has been reported [40]. Others reported observations similar to that of Cho et al. [33] from studies on patient sera of nephropathia epidemica (NE), a mild form of HFRS [41,42]. Analysis of NE patient sera using ELISA showed that while the titers of IgM reactive to PUUV Gn and nAb were low, anti-N and anti-Gc IgM ELISA titers were very high in the acute phase; IgG response was low for all three structural proteins in the acute phase but increased to a high level that lasted 2 years into convalescence when IgM responses were undetectable. IgG responses of late convalescent sera (drawn 10–20 years after onset) were similar to those at 2 years [41]. Although studies on antigen-specific antibody responses after the Hantavax vaccination or natural HTNV infection in humans are scarce, we might extrapolate from studies on other *orthohantavirus* infections that there were at least strong anti-N antibody responses after Hantavax vaccination.

Anti-N antibodies do not have neutralizing activity [43]. The neutralizing activity of anti-Gc antibodies is also low at least as acute phase IgM antibodies [41], consistent with observations of very low nAb positivity rates after the first dose of Hantavax (Table 1). However, antibody responses to Hantavax look different from natural infection. Unlike rapidly waning nAb responses after vaccination, high titers of nAb have been observed in *orthohantavirus*-exposed people tens of years after natural infection [3,41,42,44]. In natural infection, IgG responses against all three structural proteins persisted, whereas IFA positivity after vaccination suggested that all antibody responses, nAb or non-nAb, declined to a low level one year after 2 + 1 or 3 + 1 vaccination (Table 1).

The immunological background of continuous IgG production in natural *orthohantavirus* infection is not well understood, and detection of virus or viral antigen in convalescent humans has never been reported [41]. It is intriguing why this phenomenon of sustained antibody production without sustained viral antigen is not recapitulated with the inactivated virus vaccine.

### 2.4. Antigenicity of Glycoproteins

**Figure 1:**
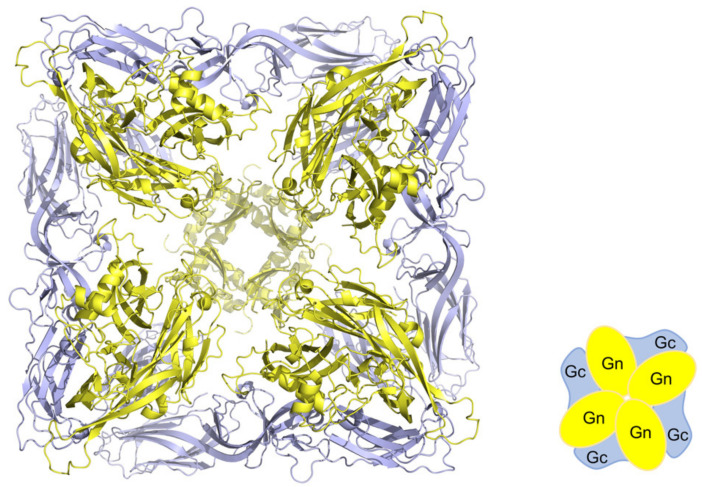
Structure of Gn/Gc tetramer. Using the atomic structural model of Andes virus spike protein (PDB ID# 6ZJM), an orthohantavirus Gn/Gc tetramer cartoon is presented on the right.

Structural studies on *orthohantavirus* membrane glycoproteins Gn and Gc [45,46] suggest differential accessibility of these proteins as an antigen. *Orthohantavirus* Gn and Gc form heterodimers which assemble into a lattice of (Gn/Gc)_4_ tetrameric spikes formed by intra-spike Gn:Gn contacts, and the surface lattice is formed by inter-spike Gc:Gc contacts (Figure 1) [46]. In this structure, Gn is in an accessible membrane distal position whereas Gc is buried. Phylogenetic analysis also suggests that Gn is subjected to the greater selective pressure of humoral immune responses [45]. Serris et al. [46] described the “breathing” spikes and called for strategy in vaccine design to expose occluded neutralizing epitopes in the spike, potentially through mutagenesis for stabilization of the pre-fusion conformation of the spike, as in the case of the respiratory syncytial virus [47].

Intriguingly, anti-Gc IgM response was much stronger than anti-Gn IgM response in the acute phase of PUUV infection. Although anti-Gc and anti-Gn IgG responses were similar during the convalescent phase, anti-Gc IgG appeared to be also dominant over anti-Gn IgG in the sera years after infection [41], which might suggest that Gc is likely highly immunogenic, but nAb binding epitopes are largely hidden [46].

### 2.5. Antigenicity of the Nucleocapsid Protein

Fast and robust anti-N antibody responses after hantavirus infection in humans are well documented [40,41,48,49]. However, anti-N antibodies are not involved in virus-neutralizing activity [43]. Is then rapid production of anti-N antibodies a byproduct of viral infection or an active strategy of immune response deviation? Interestingly, the experimental infection of ANDV in deer mice, a natural reservoir host of SNV but a heterologous host for ANDV, showed clearance signs with the appearance of anti-N antibodies without significant nAb titer [27]. Thus, anti-N antibodies might not work for the virus’s benefit. A lethal ANDV infection model of Syrian hamsters showed that vaccinating the animal with ANDV N expressing adenovirus vector offered complete protection from ANDV challenge without detectable nAb, which suggests an important role for cellular immunity [50]. A study using purified recombinant N (rN) proteins of PUUV, Topografov virus (TOPV), ANDV, and DOBV to vaccinate the bank vole showed broad cross-stimulation of lymphocytes from animals vaccinated with rN proteins in the presence of PUUV N protein. Challenging the bank voles with PUUV showed 100%, 100%, 37%, and 70% protection using vaccinations of rN proteins of PUUV, TOPV, ANDV, and DOBV, respectively, although amino acid sequence identities of the N proteins were 100%, 87%, 73%, and 60%, respectively. This study also showed that all rN proteins gave rise to antibodies cross-reactive to recombinant and native PUUV N proteins, but antibody responses against the N protein were not the major contributor of cross-protection [51]. A study using the rN protein of DOBV also showed complete protection of mice after three vaccinations and challenge, which was determined by the lack of anti-Gn or anti-Gc antibody responses [52]. By detecting viral persistence using the appearance of anti-Gn or anti-Gc antibodies as an indicator, protection via cell-mediated immune responses appear to have been assumed. Although these studies did not use a lethal animal model, it is important to note that vaccination using N protein alone could be protective mainly through N-specific T cell responses.

### 2.6. Role for Non-Neutralizing Antibody Responses

**Figure 2:**
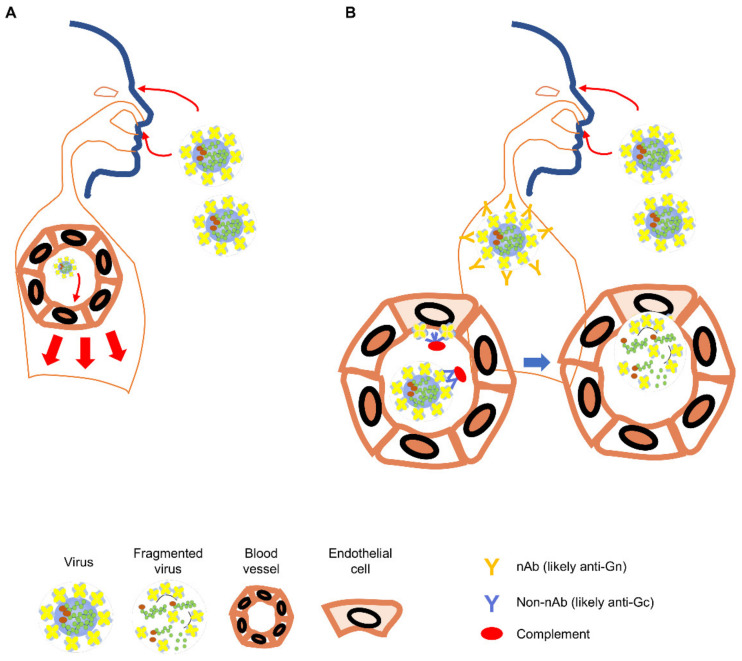
*Orthohantavirus* infection in the naïve and the Hantavax-vaccinated. (**A**) In the naïve individual, infectious viral particles entering through eyes, airways, or open skin may infect endothelial cells in the local blood vessels (light red arrows) and spread to other parts of the body (heavy red arrows) [61]. (**B**) In the Hantavax-vaccinated individual, the entering viral particles might be neutralized by nAb. Non-nAb in the vaccinated may work through the Fc function to destroy the virus particle or infected cells leading to the next phase of immune responses targeting internal proteins of the virus (blue arrows). Blood vessels are exaggerated to show virus infection of the endothelial cells and immune responses in the vaccinated after infection.

**Figure 3:**
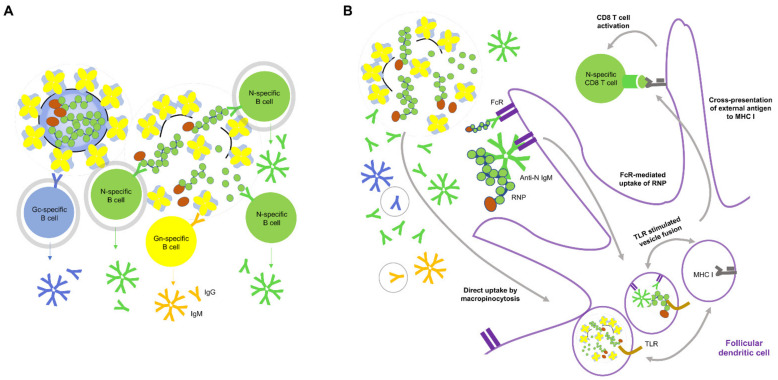
Conceptual mechanism of generation of anti-N antibody by Hantavax vaccination and anti-N antibody mediated protection. (**A**) siIWV-like Hantavax might induce more T cell-dependent or independent N-specific B cell activation than Gn- or Gc-specific B cell activation, due to a greater number of N monomer or RNP than Gn or Gc as a tetramer or membrane-embedded. B cell interactions with the antigens that can induce T cell-independent B cell activation are circled grey. (**B**) Potential mechanism of CD8 T cell memory generation by siIWV-like Hantavax: the participation of anti-N antibody in cross-presentation of external antigen on MHC I for CD8 T cell activation overlaps with that of anti-N antibody from rN vaccination. Antibodies produced by antigen-specific B cells are represented by the same color code. The antibody species in a light grey circle may not be present in phase 2 of the recall response of rN vaccination (see Figure 3). Regular external antigen presentation on MHC II for CD4 T cell activation is omitted here for brevity. The components in the cartoon are not to the scale. Refer to the legends in Figure 3 for objects not specified.

We might draw some relevance to Hantavax VE from the rN protein immunization studies. To understand Hantavax VE and protection mediated by N-specific T cell responses after rN protein immunization [51,52], we schematically reconstructed potential antibody responses from Hantavax and rN protein vaccinations and subsequent infections (Figure 2 and Figure 3, Table 2). This possibly oversimplified reconstruction is based on the generally accepted paradigm of antibody responses after infection or vaccination and the recall responses assuming equal accessibility to exposed target antigens [53,54,55,56,57,58,59,60].

Hantavax is an inactivated whole virus (IWV) vaccine, but there could be fragmentations such as split virus (SV) vaccines. We considered these possibilities of intact IWV (iIWV) and semi-intact IWV (siIWV), respectively (Table 2). A large gap between nAb positivity rate (low) and IFA or ELISA positivity rate (high) one month after the Hantavax vaccination (Table 1) suggests strong non-nAb production. If Hantavax contained SV-like (SVL) structures, there could be an additional response (Table 2). With the co-existence of iIWV and SVL, rapid T cell-independent B-cell activation and antibody secretion could occur for N as well as Gn and Gc. If the composition of Hantavax were only 50% iIWV (iIWV:SVL = 1:1), the number of N-containing particles could be three times that of Gn- and Gc-containing iIWV particles since there are three N-coated genome ribonucleoprotein (RNP) complexes per virion. The number of N-containing particles could be six times more abundant than each Gn- or Gc-specific B-cell receptor (BCR)-bearing B cell-bound iIWV, which would be commensurate with the assumption of equal antigenic epitope accessibility of the T cell-independent N-specific antibody response up to 6-fold of the T cell-independent Gn- or Gc-specific antibody response. However, although the term SVL is used to emphasize the possible presence of RNP outside the vaccine virus particle in Hantavax, it might be more like RNP plus a large viral membrane fragment containing multiple Gn/Gc tetramers rather than RNP plus soluble Gn/Gc tetramers. In this case, T cell-independent N-specific antibody response might be up to 3-fold of the T cell-independent Gn- or Gc-specific antibody response. Additionally, even when T cell-dependent B cell activation is considered against antigens including monomeric or oligomeric complexes of Gn, Gc, and N, the number of antigenic particles for N-specific B cells is likely higher than for Gn- and Gc-specific B cells, since there tends to be a lot more nucleocapsid proteins than viral envelope proteins (Figure 3A) [62,63]. More N-specific antibodies would enhance N uptake by Fc receptor (FcR)-bearing antigen-presenting cells, such as follicular dendritic cells (FDC) [59,60]. More N-specific antibody-mediated uptake by FcR bearing antigen-presenting cells would also enhance the activation of N-specific CD8 T cells and the generation of CD8 memory T cells through antigen cross-presentation (Figure 3B). Furthermore, anti-N antibody-mediated uptake of RNP would be better cross presented on MHC I due to the possibility of RNA in the RNP stimulating the Toll-like receptors (TLR) [58]. The macropinocytosis pathway of antigen uptake of Figure 3B could have been directly utilized for CD8 T cell memory generation in siIWV-like Hantavax vaccinated, where RNP as the stimulator of TLR must have been the reason for more N-specific CD8 T cell memory generation. Gn and Gc could freeload with RNP in a macropinocytosis vesicle or in FcR-mediated uptakes and be presented on MHC I by RNP stimulating TLR, but the situation of Gn and Gc cross-presentation on MHC I could be rather precarious, compared with the independence of RNP. If rN vaccine contained endosomal TLR stimulating adjuvant, the macropinocytosis pathway could be used similarly for N-specific CD8 T cell memory generation as siIWV-like Hantavax. If Hantavax had generated a strong N-specific antibody response, it would likely have been due to the co-existence of iIWV and SVL particles, which could also have opened a path for CD8 T cell memory generation (Figure 3B). N-specific antibodies, although non-neutralizing, could indicate the presence of protective N-specific CD4 and CD8 T cell memory that could be recalled upon HTNV infection.

Our schematic analysis may oversimplify the actual immune responses upon Hantavax vaccination. However, we can see that anti-N antibodies might not be just non-nAb. During acute HTNV infection, strong N-specific T cell responses measured by interferon-g (IFN-g) enzyme-linked immunosorbent spot analysis were associated with mild or moderate HFRS [64]. In the case of the rN protein vaccination, as summarized in Table 2, there could be N-specific antibody response and the generation of N-specific memory B cells and CD4 memory T cells, and N-specific CD8 memory T cells as well, depending on the presence of endosomal TLR stimulating adjuvant in the vaccine (Figure 3B). Since there were no memory B cells for anti-Gn and anti-Gc antibodies to function as nAb upon infection, protection by the rN protein vaccination must have depended heavily on N-specific memory T cells rather than anti-Gn and anti-Gc nAb [51]. Indeed, anti-Gn and anti-Gc antibodies were considered only as signs of replication enough to give rise to naïve B cell antibody responses—signs of no protection.

Interestingly, while mice immunized with DNA vaccine encoding the secreted form of N protein of PUUV showed not only N-specific antibody response but also N-specific lymphoproliferative response, mice immunized with purified N protein showed only high N-specific antibody response without lymphoproliferative response [65], which is in disagreement with another similar study using rN [51]. It is unknown whether this discrepancy was a matter of using full-length rN antigen [51] or using only N-terminal 117 amino acids of N [65]. Nicacio et al.’s study [51] showing overall sequence identity not directly correlated with cross-protectivity suggests the importance of sharing the immunodominant region’s sequence for cross-protective T cell-mediated immune response. There could have been other multiple factors involved, including factors as simple as the amount of antigen. Further study might resolve the exact mechanism of this discrepancy.

Orthohantavirus infection under naïve conditions would generate antibody responses initially like those presented in Table 2. As soon as a host begins to remove viral particles and virus-infected cells and disintegrated virus and/or virus-infected cells appear, antibody responses like those of siIWV vaccinated might kick in, and N-specific responses might dominate (Figure 3A). Viral genomic remnants appear to be present long after viable viruses can be detected [66,67]. We speculate that the intriguing observation of strong acute phase antibody response to N, an internal protein, similar to or even stronger than the membrane surface proteins Gn and Gc, might be explained by this conceptualization.

### 2.7. Strategy to Design Orthohantavirus Vaccines as Pandemic Preparedness

Designing a viral vaccine in preparation for the future necessitates broad-spectrum cross-reactivity that is effective for decades. Which of the proteins of *orthohantavirus* could be best as a vaccine target antigen in designing a broad-spectrum *orthohantavirus* vaccine? While broad cross-reactivity in antibody responses to N proteins was observed [51,68,69,70,71,72], Gc cross-reactivity was weak, and Gn cross-reactivity was weakest [68]. However, DNA vaccination of the M gene of HTNV or SEOV protected hamsters from the challenge of HTNV, SEOV, and DOBV, although not PUUV [73]. What could have been the mechanism of this M gene-mediated cross-protection? Although the discovery of an urban rat virus later named SEOV was based on rat sera’s reactivity in IFA with HTNV [74,75], the SEOV Gn was not reactive with anti-HTNV serum, and Gc was only very weakly reactive. Strong reactivity of SEOV N with anti-HTNV serum suggests the major contribution of anti-N antibody to rat sera reactivity with HTNV in the IFA [68]. Although the generation of cross-reactive nAb using HTNV or SEOV Gn- and Gc-expressing DNA vaccination has been reported [73], it was rather weak. Attempts to combine M genes of different viruses in a DNA vaccine appear promising [76,77,78], but the ultimate concern is whether Gn and Gc would be the same in *orthohantavirus* threats that may appear decades later.

We have discussed the potential role for non-nAb, N-specific antibodies in enhancing N-specific T cell responses, schematically analyzing existing studies. It is not known whether *orthohantavirus* could be neutralized intracellularly by anti-N antibodies as seen in intracellular neutralization of rotavirus by anti-VP6 antibodies [79]. The RNP of enveloped *orthohantavirus* is not likely exposed in endosomes, where intracellular antibodies are known to function, unlike the nucleocapsid protein VP6 of non-enveloped rotavirus. Since anti-N antibodies were shown not to have a neutralizing activity in the in vitro infection assay [43], a role for anti-N antibodies needs to be investigated in an in vivo context. There is a report that mice vaccinated with recombinant NP (rNP) of influenza virus plus LPS could be protected from influenza virus challenge [80]. In this study, vaccination induced high titers of anti-NP antibodies in the serum but limited NP-specific CD8 T cell responses. Interestingly, transfer of rNP immune sera could protect the recipient mice from influenza virus challenge only when the T cell functions of the recipient mice were intact. The requirement of functional T cells for protection by anti-NP antibodies appears to overlap with our conceptualization of anti-N antibody function in Figure 3B. The poor NP-specific CD8 T cell response after vaccination in this study might have been due to using LPS as an adjuvant, which binds to TLR4 in the plasma membrane, not in the endosome [81]. Coincidentally, the influenza virus is similar to *orthohantavirus* in terms of being an enveloped virus containing a segmented single-stranded RNA genome [82]. However, many of the mechanisms of non-nAb antibody and T cell responses suggested in other viruses have not been proven experimentally yet for *orthohantavirus* and its specific vaccine types.

Broad cross-reactivity in antibody responses to the N protein might be utilized for broad-spectrum vaccine development [51,69,70,71,72]. However, the non-nAb N-specific antibody-mediated protective immune responses should be characterized through further studies. N-expressing DNA or mRNA vaccines and the differential effects of the secreted form of N and intracellular N need to be better characterized. Conceptually, (1) rN protein vaccination would generate both CD4 and CD8 T cell memory in the presence of cross-presentation; (2) DNA or mRNA expression of secreted N would generate both CD4 and CD8 T cell memory without the need for cross-presentation; (3) DNA or mRNA expression of intracellular N would mainly support CD8 T cell memory generation. These concepts need to be tested also. Similar concepts of conserved NP-specific response could also be applied to designing a universal vaccine against influenza virus, containing eight genome RNP complexes per virion [82]. In the case of influenza viruses, due to strong nAb responses against their surface glycoprotein hemagglutinin [83,84], the role for NP-specific non-nAb responses facilitating resistance to influenza virus has been largely disregarded [80]. The question of why people get repeatedly infected with antigenically drifted influenza virus strains is part of the reason why the possibility of anti-NP antibody-mediated protection against influenza is disregarded. It is difficult to find studies looking into the possibility of reduced severity of the disease in the case of repeated infections of influenza virus compared with a first-time infection. A study assessing the efficacy of a T-cell-based influenza vaccine targeting NP and M1 in volunteers showed reduced virus shedding and reduced symptoms compared with the control [85]. This study equated the reduction level to 60% vaccine efficacy, a similar level for inactivated influenza vaccines shown when the circulating strain and the vaccine strain matched. An open discussion may be needed about a balance between broad range reduction of disease severity and limited range of absolute protection in weighing the benefits of broad-spectrum non-nAb and specificity-limited nAb.

The caveat of *orthohantavirus* vaccine studies may be animal models in which *orthohantavirus* replication is not highly active. When the viral growth rate is low, it is easy to clear the infection. The issue is whether reported protective effects in such animals would be translatable to humans. Instead of using asymptomatic animals [9] and surrogate detection of viral persistence by the presence of anti-Gn or anti-Gc antibodies, the lethal animal model of ANDV infection in the Syrian hamster [86] might be better utilized to study true cross-protection after immunization using IWV, SV, a recombinant protein or antigen-expressing DNA, or mRNA.

## 3. Conclusions and Perspective

We have discussed the antigenicity of Hantavax and potential *orthohantavirus* vaccines. Strong anti-N antibody induction due to Hantavax vaccination encompasses the effect of rN vaccination in addition to the IWV and SV vaccination effects. The current study trend might be towards subunit vaccines using purified proteins or structural protein-expressing DNA vaccines, but whole virus vaccines or split vaccines, including Hantavax, also need to be better characterized. It appears that further analysis of the immune responses of Hantavax using animal models would enlighten us on many aspects. A Hantavax cross-reactivity study using the Syrian hamster model of ANDV infection would be valuable for determining the potential of Hantavax as one existing pandemic preparedness vaccine against diverse *orthohantaviruses*. Although Hantavax has shown only short-term efficacy, a pandemic vaccine might not necessarily need to be proven for long-term efficacy. In the event of an *orthohantavirus* pandemic, the priority may be proven safety and the potential to cut the chain of infection. Although not related to antigenicity, whether the Hantavax production method could meet pandemic capacity also needs to be examined to reformulate Hantavax as a pandemic vaccine.

## Figures and Tables

**Table 1:** Antibody responses after the Hantavax vaccination.

				Serum Antibody Positivity (%) at a Given Time Point after Vaccination (Month) ^b^											
Study	No. of Study Participants ^a^	Vaccine Dosage	Test Method	Day 0	1	2	3	12	13	14	15	17	25	37	49
ref. 33	64 −> 14	3	Neutralization	**0**	**13**	75		**14.2**	50						
			IFA	**0**	**79**	97		**37**	94						
ref. 34	30 −> 30	2	Neutralization	**0**	**3.3**	16.7									
			^c^ Neutralization +	**0**	**6.7**	33.3									
			ELISA	**0**	**46.7**	76.7									
ref. 35	142 −> 64	3	Neutralization	**0**	**n.d.**	23.24			**1.41**	45.07			40.63	15.63	12.5
			IFA	**0**	**n.d.**	90			**10.56**	87.32			34.68	17.74	10.48
ref. 36	289 −> 277	4	Neutralization	**0**	**n.d.**	**40.97**	72.32		**7.61**	55.71	41.91	27.44			
			IFA	**0**	**n.d.**	**83.03**	92.81		**21.22**	95.68	67.16	51.13			

^a^ the number of participants at the start and the end of the study; ^b^ each shaded column and bold letter indicate vaccination time points; ^c^ rate of neutralizing antibody positivity with the supplementation of 5% normal human sera in the assay; IFA, immunofluorescent assay; ELISA, enzyme-linked immunosorbent assay; n.d., not determined.

**Table 2:** Conceptual antibody responses after vaccination and recall responses upon infection.

Vaccine	Immune Responses after Vaccination	Recall Responses after Virus Infection
Hantavax (iIWV ^a^)	Multiple Gn/Gc on iIWV lead to T cell-independent B cell activation for anti-Gn and anti-Gc IgM production. T cell-dependent anti-Gn/Gc response : Gn- and Gc-specific B cells take up iIWV, present Gn, Gc, and N on MHC II, and get help for anti-Gn and anti-Gc production from Gn, Gc, and N-specific T cells prior activated by FDC that had taken up iIWV, processed and presented antigens on MHC II for CD4 T cells.	Phase 1 (live virus antigen) : anti-Gn and anti-Gc recall response involving Gn- and Gc-specific memory T and B cells, in addition to T cell-independent B cell activation for anti-Gn and anti-Gc IgM production. Phase 2 (SVL ^b^ antigen from disintegrated virus and infected cell by the Fc-mediated activity of anti-Gn and anti-Gc) : T cell-independent anti-N IgM against released RNPs
Hantavax (semi-iIWV)	Multiple Gn/Gc on iIWV and multiple N on RNP of SVL lead to T cell-independent B cell activation for anti-Gn, anti-Gc, and anti-N IgM production. T cell-dependent anti-Gn, anti-Gc, and anti-N response : Gn-, Gc-, and N-specific B cells take up antigen from siIWV, present Gn, Gc, and N on MHC II, and get help for anti-Gn, anti-Gc, and anti-N production from Gn, Gc, and N-specific T cells prior activated by FDC that had taken up siIWV, processed and presented antigens on MHC II for CD4 T cells	Phase 1 (live virus antigen) : anti-Gn and anti-Gc recall response involving Gn- and Gc-specific memory T and B cells, in addition to T cell-independent B cell activation for anti-Gn and anti-Gc IgM production. Phase 2 (SVL antigen from disintegrated virus and infected cell by the Fc-mediated activity of anti-Gn and anti-Gc) : anti-N recall response against released RNP and/or N involving N-specific memory T and B cells, in addition to T cell-independent B cell activation for anti-N IgM.
Recombinant N vaccine (rN)	No T cell-independent anti-N response T cell-dependent anti-N response : N-specific B cells take up rN, present N on MHC II, and get help from N -specific T cells prior activated by FDC that had taken up rN, processed and presented N on MHC II for CD4 T cells.	Phase 1 (live virus antigen) : no anti-N recall response. T cell-independent B cell activation for anti-Gn and anti-Gc IgM production. Phase 2 (antigen from disintegrated virus and infected cell) : anti-N recall response against released RNPs and/or N involving N-specific memory T and B cells, in addition to T cell-independent B cell activation for anti-N IgM.

^a^ iIWV, intact inactivated whole virus; ^b^ SVL, spilt vaccine-like.

## Data Availability

Not applicable.
